# Association between the composite dietary antioxidant index and chronic rhinosinusitis burden: the potential mediating role of insulin resistance

**DOI:** 10.3389/fnut.2026.1908084

**Published:** 2026-08-31

**Authors:** Hesong Wang, Feng Jin, Liang Tian, Guizhi Kang, Yingjie Yang, Ziyu Zhao, Nuo Zhang, Xue Liu, Yongning Huang

**Affiliations:** Department of Otorhinolaryngology–Head and Neck Surgery, Huludao Central Hospital, Huludao, Liaoning, China

**Keywords:** chronic rhinosinusitis, composite dietary antioxidant index, HOMA-IR, insulin resistance, Lund-Mackay score, mediation analysis, triglyceride-glucose index

## Abstract

**Background:**

Chronic rhinosinusitis (CRS) is a heterogeneous inflammatory sinonasal disease with variable radiologic and symptom burden. Dietary antioxidant capacity and insulin resistance may be related to CRS burden, but their interrelationship remains unclear. This study investigated the association of energy-adjusted composite dietary antioxidant index (CDAI) with CRS burden and explored the potential mediating roles of insulin resistance-related indicators.

**Methods:**

In this single-center cross-sectional study, we analyzed baseline data from 340 adult patients with clinically diagnosed CRS who were recruited at Huludao Central Hospital. Dietary intake was assessed using 24-h dietary recalls, and the CDAI was calculated by summing standardized values of six energy-adjusted antioxidant nutrients. CDAI was analyzed as tertiles of the CDAI score and as standardized CDAI per 1-SD increment. HOMA-IR was specified as the primary mediator, and the triglyceride-glucose (TyG) index was considered a pragmatic non-insulin mediator. The primary outcome was Lund-Mackay CT score, and the secondary outcome was SNOT-22 score. Multivariable regression, binary sensitivity analyses, exploratory mediation analyses, and subgroup analyses were performed.

**Results:**

Higher CDAI tertiles were associated with lower fasting insulin, HOMA-IR, TyG index, and Lund-Mackay CT score. In fully adjusted models, each 1-SD increment in CDAI was associated with lower Lund-Mackay CT score (*β*, −0.799; 95% CI, −1.069 to −0.528; *p* < 0.001) and lower SNOT-22 score (*β*, −0.979; 95% CI, −1.866 to −0.093; *p* = 0.031). In exploratory mediation analyses, HOMA-IR accounted for part of the association between CDAI and Lund-Mackay CT score, with an indirect effect of −0.203 and a mediated proportion of 25.4%. TyG showed a smaller indirect component, with an indirect effect of −0.115 and a proportion mediated of 14.4%. Sensitivity and subgroup analyses showed directionally consistent findings.

**Conclusion:**

Higher CDAI was associated with lower radiologic CRS burden and modestly lower symptom burden among adults with CRS. Exploratory mediation analyses suggested that insulin resistance may partly account for the association between CDAI and radiologic disease burden, with TyG providing supportive evidence as a pragmatic non-insulin surrogate. These cross-sectional findings highlight a potential diet-metabolism relationship in CRS and support further prospective and interventional studies to clarify temporality, causality, and biological significance.

## Introduction

Chronic rhinosinusitis (CRS) is a persistent inflammatory disorder of the nasal and paranasal sinus mucosa and is generally defined by symptoms lasting at least 12 weeks together with objective evidence of mucosal inflammation on nasal endoscopy or computed tomography (CT) (1, 2). CRS affects a substantial proportion of adults worldwide; symptom-based estimates often exceed 10%, whereas estimates requiring both symptoms and objective evidence are lower but more clinically specific (3, 4). Beyond nasal obstruction, discharge, facial pressure, and olfactory dysfunction, CRS contributes to impaired sleep, reduced work productivity, repeated healthcare utilization, and diminished quality of life, making identification of modifiable systemic factors clinically relevant (5).

CRS is heterogeneous. Patients may differ by radiologic burden, nasal polyp status, eosinophilic inflammation, comorbid asthma or allergic rhinitis, and patient-reported symptom severity (1, 2). Current guideline-based frameworks increasingly emphasize inflammatory phenotypes, endotypes, and treatable traits rather than a purely local infection model (5, 6). However, most studies still focus on local inflammatory mechanisms, while the contribution of systemic nutritional and metabolic status to objective CRS burden remains insufficiently characterized.

Oxidative stress has been implicated in CRS pathophysiology through effects on epithelial barrier integrity, mucosal inflammation, and immune regulation (7, 8). Recent mechanistic studies suggest that epithelial oxidative stress, reactive oxygen species accumulation, and inflammasome activation may participate in chronic rhinosinusitis with nasal polyps (CRSwNP) inflammation and epithelial alarmin release (9, 10). These observations provide a rationale for evaluating whether habitual dietary antioxidant capacity is associated with the inflammatory and radiologic burden of CRS. Single antioxidant nutrients may not adequately capture the combined antioxidant potential of habitual diet. The composite dietary antioxidant index (CDAI) integrates multiple antioxidant micronutrients, typically including vitamins A, C, and E, carotenoids, zinc, and selenium, thereby representing a broader dietary antioxidant profile than any individual nutrient alone (11). Recent population-based studies have linked higher CDAI with more favorable cardiometabolic profiles and lower insulin resistance-related indices, suggesting a potential diet-metabolism relationship relevant to inflammatory diseases (12–14).

Insulin resistance may serve as a plausible metabolic link between dietary antioxidant status and CRS burden. It is closely linked to chronic low-grade inflammation, oxidative stress, and immune-metabolic dysregulation (15, 16). Homeostatic model assessment of insulin resistance (HOMA-IR) is a classical fasting glucose-insulin index of insulin resistance, whereas the TyG index provides a pragmatic non-insulin surrogate based on fasting triglycerides and glucose (17, 18). Recent reviews and population studies further support TyG as a simple marker of insulin resistance-related metabolic risk (19, 20). Whether insulin resistance may partly account for the association between CDAI and CRS disease burden remains unclear.

In this cross-sectional analysis of prospectively collected baseline data from adults with CRS, we aimed to investigate whether energy-adjusted CDAI was associated with objective radiologic disease burden, as assessed by Lund-Mackay CT score, and with patient-reported symptom burden, as assessed by 22-item Sino-Nasal Outcome Test (SNOT-22) score. We further performed exploratory mediation analyses to examine whether insulin resistance, assessed using HOMA-IR and the TyG index, may partly account for the association between CDAI and radiologic CRS burden.

## Methods

### Study design and participants

**Figure 1:**
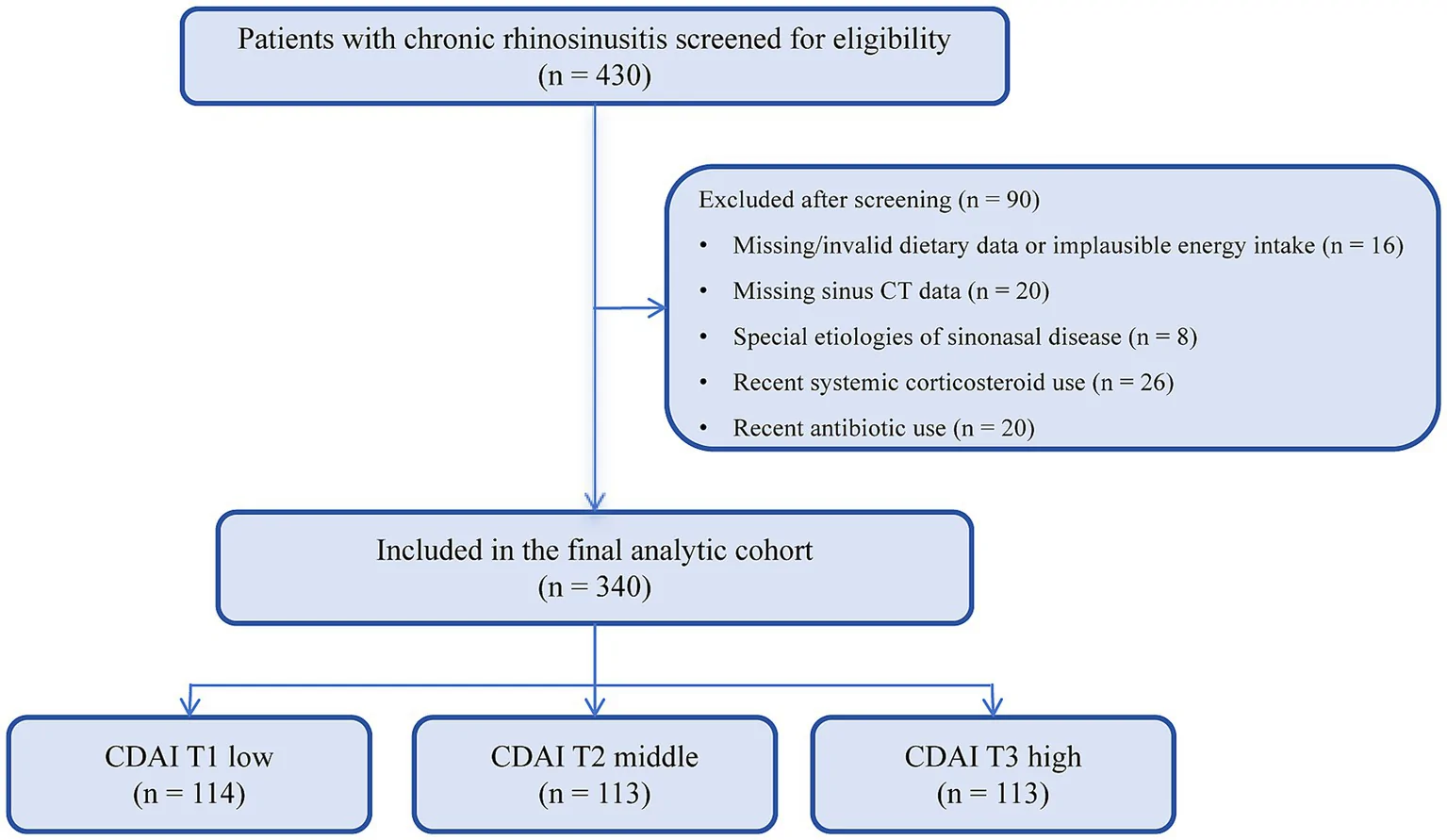
Flowchart of participant selection and CDAI tertile grouping.

This study was designed as a cross-sectional analysis of prospectively collected baseline data from adult patients with CRS who were recruited from the Department of Otorhinolaryngology–Head and Neck Surgery, Huludao Central Hospital, Liaoning, China, between January 2022 and January 2026. Eligible patients were consecutively recruited during the study period from patients undergoing baseline CRS evaluation in the department. The cohort included patients evaluated in routine clinical practice, including patients assessed for further medical management or surgical planning. CRS was diagnosed according to contemporary guideline-based principles requiring persistent sinonasal symptoms and objective evidence of sinonasal inflammation (1, 2). Reporting was guided by the Strengthening the Reporting of Observational Studies in Epidemiology (STROBE) recommendations (21). Patients were eligible if they were adults aged 18 years or older, had not undergone prior sinus surgery, met diagnostic criteria for CRS, completed dietary assessment, underwent baseline sinus CT, had fasting glucose, fasting insulin, and lipid measurements, and completed SNOT-22 assessment. Patients were excluded if they had missing or invalid dietary data, missing CT data, special etiologies of sinonasal disease, recent systemic corticosteroid use, recent antibiotic use, or clearly implausible energy intake. The participant selection process is shown in Figure 1. The study protocol was approved by Huludao Central Hospital; approval number HCHKY22011. Written informed consent was obtained from all participants.

### Assessment of energy-adjusted CDAI

Dietary intake was assessed at baseline using repeated 24-h dietary recalls. Each participant completed three 24-h dietary recalls, including two weekdays and one weekend day, to better capture weekday-weekend variation in dietary intake. The recalls were administered face-to-face by trained investigators using a standardized interview procedure. Participants were asked to report all foods and beverages consumed during the preceding 24 h, including food type, preparation method, portion size, and meal timing. Standardized household measures, food portion-size reference pictures, and commonly used utensils were used to assist portion-size estimation.

Nutrient intakes were calculated using the Chinese Food Composition Tables, 6th edition. For each participant, the average daily intake of total energy and antioxidant micronutrients across the three recalls was used for analysis. Implausible total energy intake was defined *a priori* as <800 or >4,200 kcal/day for men and <500 or >3,500 kcal/day for women, and participants with implausible energy intake were excluded from the analytic sample. Micronutrients derived from dietary supplements were not included in the CDAI calculation, because CDAI was intended to reflect antioxidant intake from foods and beverages. Dietary supplement use was recorded and further addressed in sensitivity analyses by excluding supplement users.

The CDAI was constructed from six antioxidant nutrients: vitamin A, vitamin C, vitamin E, carotenoids, zinc, and selenium (12). Before standardization, each antioxidant nutrient was energy-adjusted using the residual method. Briefly, each nutrient intake was regressed on total energy intake, and the residual from this regression was added to the study population mean intake of that nutrient to obtain the energy-adjusted nutrient value. The energy-adjusted values were then standardized within the current study population using the study-specific mean and standard deviation. For each nutrient, a z-score was calculated as follows: *z* = (energy-adjusted nutrient value—study population mean)/study population standard deviation. The CDAI score was calculated as the sum of the six study-specific standardized nutrient *z*-scores, with higher values indicating greater dietary antioxidant capacity. No external reference distribution was used for CDAI standardization. CDAI was analyzed in two forms. CDAI tertiles were defined using the CDAI score, whereas standardized CDAI was generated by rescaling the CDAI score to a mean of 0 and a standard deviation of 1 for continuous regression and mediation analyses. In the final analytic sample, the CDAI tertile cutoffs were as follows: T1, CDAI score ≤ − 2.327; T2, −2.327 < CDAI score ≤ 2.672; and T3, CDAI score > 2.672. Estimates for continuous analyses are reported per 1-SD increment in standardized CDAI.

### Assessment of insulin resistance-related measures

Two insulin resistance-related indicators were evaluated. HOMA-IR was specified as the primary mediator in the exploratory mediation analyses and was calculated as fasting plasma glucose (mmol/L) multiplied by fasting insulin (μU/mL), divided by 22.5 (17). TyG was calculated as ln[triglycerides (mg/dL) × fasting plasma glucose (mg/dL)/2] and was considered a pragmatic non-insulin mediator (19). Although fasting plasma glucose and triglycerides are reported in mmol/L in the descriptive tables, both variables were converted to mg/dL using standard conversion factors before TyG calculation. Fasting plasma glucose, fasting insulin, triglycerides, and derived insulin resistance-related indicators were rechecked against the analytic dataset, and no truncation or winsorization was applied to these variables.

### Outcomes

The primary outcome was Lund-Mackay CT score, a radiologic staging score ranging from 0 to 24, with higher values indicating greater sinus opacification and radiologic disease burden (22). Sinus CT scans were obtained as part of the baseline clinical assessment. Dietary assessment, fasting blood sampling, SNOT-22 assessment, and sinus CT were completed on the same baseline assessment day. Lund-Mackay CT scores were independently assessed by two senior otolaryngologists using the standard Lund-Mackay scoring system. The raters did not have access to dietary intake, CDAI tertile, fasting glucose, fasting insulin, lipid measurements, HOMA-IR, or TyG index during CT scoring. If the two raters assigned the same score, that score was used for analysis. In cases of disagreement, a third senior otolaryngologist independently reviewed the CT scan, and the final score was determined after adjudication. The highest tertile of Lund-Mackay score was defined as high Lund-Mackay burden for binary sensitivity analyses.

The secondary outcome was SNOT-22 score, assessed using a validated Chinese version of the 22-item Sino-Nasal Outcome Test. SNOT-22 is a disease-specific patient-reported outcome measure ranging from 0 to 110, with higher scores indicating worse symptoms and quality-of-life impairment (23, 24). The highest tertile of SNOT-22 score was defined as high SNOT-22 burden for sensitivity analyses. SNOT-22 was not used as a mediation outcome in the main analysis because it is influenced by multiple symptom, psychological, sleep, and quality-of-life dimensions beyond radiologic inflammation.

### Covariates

Potential confounders were selected *a priori* based on clinical relevance and prior literature. Demographic and lifestyle factors included age, sex, body mass index (BMI), current smoking, current alcohol use, and total energy intake. CRS-related clinical factors included allergic rhinitis, asthma, CRSwNP phenotype, and intranasal corticosteroid use. Metabolic comorbidities included diabetes and hypertension. Operational definitions were prespecified using baseline interview data, medical records, and available clinical measurements. Current smoking and current alcohol use were defined as self-reported current active smoking and current alcohol drinking at baseline, respectively. Dietary supplement use was defined as self-reported use of vitamin, mineral, antioxidant, or other dietary supplements during the baseline dietary assessment period. Diabetes was defined as a documented physician diagnosis, antidiabetic medication use, or fasting plasma glucose ≥7.0 mmol/L. Hypertension was defined as a documented physician diagnosis, antihypertensive medication use, or baseline blood pressure ≥140/90 mmHg when available. Intranasal corticosteroid use was defined as documented use of an intranasal corticosteroid spray for CRS before enrollment. Waist circumference, HDL-C, and selected metabolic variables were described in baseline tables but were not all included in the primary multivariable model to reduce over adjustment of insulin resistance-related metabolic pathways and redundancy with mediator-related variables. The fully adjusted model was prespecified to include key demographic, lifestyle, CRS-related, and cardio metabolic covariates. BMI, diabetes, and hypertension were included to account for general cardio metabolic status, whereas CRSwNP phenotype, allergic rhinitis, asthma, and intranasal corticosteroid use were included to account for CRS phenotype, comorbidity, and treatment status. Dietary supplement use and antidiabetic drug use were evaluated in restricted sensitivity analyses, and prior sinus surgery was handled as an exclusion criterion. To support covariate selection, supplementary metabolic-variable diagnostics were performed, including selected Spearman correlations, variance inflation factors in an expanded model additionally including waist circumference and HDL-C, and an expanded-adjustment sensitivity model. Detailed definitions, formulas, and analytical roles of the main study variables are summarized in Supplementary Table S1.

CRS phenotype was classified as CRSwNP or CRSsNP according to nasal endoscopic findings and sinus CT assessment. Disease duration was defined as the interval between the onset of CRS-related symptoms and baseline enrollment. Medication exposure before enrollment, including antihistamine use, leukotriene modifier use, biologic therapy, and long-term intranasal corticosteroid use, was obtained from medical records and baseline clinical interviews. Peripheral eosinophil counts were recorded as a systemic inflammatory marker and summarized across CDAI tertiles. Elevated peripheral eosinophils were defined according to the institutional laboratory reference range. Standardized tissue eosinophil counts and standardized endoscopic scores were not systematically available for all participants; therefore, these variables were described as unavailable for formal endotype classification or endoscopic score-adjusted analysis. Additional clinical and treatment characteristics, including disease duration, antihistamine use, leukotriene modifier use, and biologic therapy, were summarized descriptively to characterize cohort heterogeneity. The primary multivariable models retained the prespecified CRS-related covariates, including CRSwNP phenotype, allergic rhinitis, asthma, and intranasal corticosteroid use.

### Statistical analysis

The final analytic cohort included 340 complete cases with available exposure, outcome, mediator, and covariate data required for the primary analyses. Baseline characteristics were summarized according to CDAI tertiles. Continuous variables are presented as mean ± standard deviation, and categorical variables are presented as *n* (%). Between-group comparisons used analysis of variance or nonparametric tests for continuous variables and chi-square tests for categorical variables as appropriate.

Multivariable linear regression was used to estimate associations of CDAI with continuous Lund-Mackay and SNOT-22 scores. CDAI was analyzed both as tertiles of the CDAI score and as standardized CDAI, defined as the CDAI score rescaled to a mean of 0 and a standard deviation of 1. Continuous regression estimates are reported per 1-SD increment in standardized CDAI, with the lowest CDAI tertile used as the reference for tertile-based analyses. P for trend was calculated by modeling CDAI tertile as an ordinal variable. Model 1 adjusted for age and sex. Model 2 further adjusted for BMI, current smoking, current alcohol use, and total energy intake. Model 3 additionally adjusted for allergic rhinitis, asthma, CRSwNP phenotype, diabetes, hypertension, and intranasal corticosteroid use.

Exploratory mediation analyses were performed to examine whether HOMA-IR and TyG partly accounted for the association between CDAI and Lund-Mackay score. Standardized CDAI was treated as the exposure, each insulin resistance-related indicator was evaluated separately as the mediator, and Lund-Mackay score was treated as the outcome. HOMA-IR and TyG were modeled in separate mediation models rather than in a joint mediator model because they are correlated metabolic indices that reflect overlapping but not identical aspects of insulin resistance-related metabolism. The mediator model and outcome model included the same covariates as the fully adjusted model, including age, sex, BMI, current smoking, current alcohol use, total energy intake, allergic rhinitis, asthma, CRSwNP phenotype, diabetes, hypertension, and intranasal corticosteroid use. For each mediator, the mediator model regressed the mediator on standardized CDAI and covariates, and the outcome model regressed Lund-Mackay score on standardized CDAI, the mediator, and the same covariates. Exposure-mediator interaction terms were additionally tested by including the standardized CDAI × mediator interaction in the outcome model. Because no statistically significant exposure-mediator interaction was observed, the final mediation models were fitted without interaction terms. Indirect effects, direct effects, total effects, and mediated proportions were estimated using nonparametric bootstrap procedures with 1,000 resamples. Bootstrap percentile 95% confidence intervals were reported (25). CDAI was expressed per 1-SD increment, whereas HOMA-IR, TyG, and Lund-Mackay score were retained on their original analytical scales; therefore, the reported mediation effects and pathway coefficients are unstandardized estimates except for the standardized CDAI exposure. Given the cross-sectional design, these mediation analyses were interpreted as exploratory and were not intended to establish temporal or causal mediation. Because SNOT-22 was a secondary patient-reported outcome with broader determinants, mediation analyses were restricted to Lund-Mackay score.

**Figure 2:**
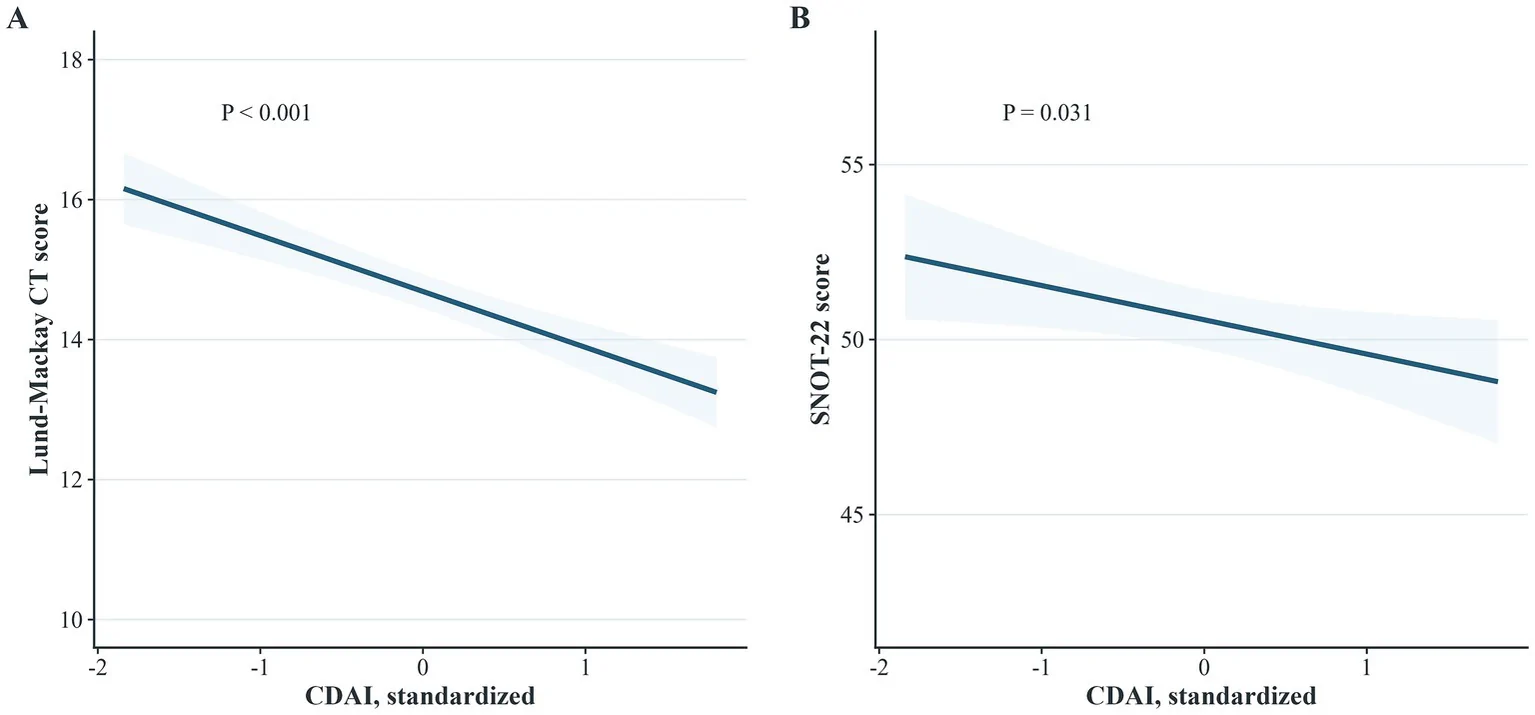
Adjusted associations of standardized CDAI with continuous CRS burden outcomes. Panel **(A)** shows the adjusted association between CDAI and Lund-Mackay CT score. Panel **(B)** shows the adjusted association between CDAI and SNOT-22 score. Shaded areas represent 95% confidence intervals.

Sensitivity analyses evaluated associations with high Lund-Mackay burden and high SNOT-22 burden, defined by the upper tertile of each score distribution in the final analytic sample. The thresholds were Lund-Mackay CT score ≥16.006 and SNOT-22 score ≥53.508. If tied values occurred at the cutoff, all participants with the cutoff value were assigned to the same category. Restricted analyses were performed after excluding patients with diabetes, antidiabetic drug use, dietary supplement use, current smoking, and intranasal corticosteroid use. Subgroup analyses were conducted by age, sex, BMI, smoking, alcohol use, CRS phenotype, allergic rhinitis, asthma, diabetes, hypertension, and intranasal corticosteroid use. Subgroup and sensitivity analyses were conducted as supportive analyses to evaluate robustness and consistency rather than as separate confirmatory hypothesis tests. Therefore, no formal multiple testing correction was applied to these analyses, and their *p* values were interpreted as nominal. Interpretation of subgroup and sensitivity analyses emphasized effect estimates, 95% confidence intervals, interaction *p* values where applicable, and consistency of direction across analyses. For the primary analyses, two-sided p values <0.05 were considered statistically significant. To formally assess potential nonlinearity in Figure 2, restricted cubic spline models were fitted for standardized CDAI with three knots at the 10th, 50th, and 90th percentiles, using the same covariates as the fully adjusted model. Model diagnostics for the fully adjusted linear regression models included assessment of residual distribution, heteroscedasticity, and influential observations. Analyses were conducted using R software (version 4.4.3). Exploratory mediation analyses were performed using the mediate() function in the R package “mediation.”

## Results

### Participant selection and baseline characteristics

A total of 430 patients with CRS were screened. After excluding patients with missing or invalid dietary data, missing CT data, special etiologies, recent systemic corticosteroid use, or recent antibiotic use, 340 patients were included in the final analysis (Figure 1).

Baseline characteristics by CDAI tertiles are shown in Table 1. Age, BMI, sex distribution, current smoking, current alcohol use, allergic rhinitis, asthma, diabetes, hypertension, and intranasal corticosteroid use were generally balanced across CDAI tertiles. Higher CDAI was accompanied by lower fasting insulin, triglycerides, HOMA-IR, and TyG index, whereas fasting plasma glucose was similar across groups. The mean Lund-Mackay CT score decreased from 15.58 in T1 to 13.73 in T3, and high Lund-Mackay burden decreased from 45.6 to 20.4%. SNOT-22 score showed a directionally lower pattern across CDAI tertiles but did not differ significantly in unadjusted group comparisons. Energy-adjusted antioxidant nutrient intakes across CDAI tertiles are summarized in Supplementary Table S2. Intakes of vitamin A, vitamin C, vitamin E, carotenoids, zinc, and selenium increased progressively from T1 to T3, while total energy intake was comparable across tertiles. The overall distributions of CDAI and its components are provided in Supplementary Table S3.

**Table 1:** Baseline characteristics according to CDAI tertiles.

Variable	Overall	T1 low	T2 middle	T3 high	*P* value
Demographic and lifestyle characteristics					
Age, years	45.2 ± 11.1	45.4 ± 12.1	45.7 ± 10.4	44.5 ± 10.6	*p* = 0.676
BMI, kg/m^2^	25.01 ± 2.73	25.10 ± 2.80	24.98 ± 2.79	24.96 ± 2.63	*p* = 0.918
Waist circumference, cm	95.32 ± 5.35	96.02 ± 5.73	94.78 ± 5.18	95.14 ± 5.08	*p* = 0.197
Male sex	202 (59.4)	67 (58.8)	73 (64.6)	62 (54.9)	*p* = 0.325
Current smoking	148 (43.5)	55 (48.2)	40 (35.4)	53 (46.9)	*p* = 0.101
Current alcohol use	200 (58.8)	71 (62.3)	69 (61.1)	60 (53.1)	*p* = 0.313
Dietary supplement use	45 (13.2)	10 (8.8)	19 (16.8)	16 (14.2)	*p* = 0.190
Dietary and metabolic characteristics					
Total energy intake, kcal/day	2108.18 ± 361.34	2079.80 ± 339.40	2165.19 ± 388.52	2079.80 ± 350.88	*p* = 0.122
Fasting plasma glucose, mmol/L	5.39 ± 0.27	5.40 ± 0.29	5.40 ± 0.27	5.38 ± 0.26	*p* = 0.735
Fasting insulin, μU/mL	8.78 ± 2.04	10.07 ± 1.80	8.62 ± 1.76	7.64 ± 1.77	*p* < 0.001
Triglycerides, mmol/L	1.24 ± 0.31	1.38 ± 0.33	1.21 ± 0.28	1.14 ± 0.28	*p* < 0.001
HDL-C, mmol/L	1.17 ± 0.11	1.15 ± 0.12	1.17 ± 0.11	1.19 ± 0.10	*p* = 0.016
HOMA-IR	2.10 ± 0.48	2.41 ± 0.43	2.06 ± 0.40	1.82 ± 0.42	*p* < 0.001
TyG index	8.55 ± 0.25	8.66 ± 0.25	8.53 ± 0.23	8.47 ± 0.24	*p* < 0.001
CRS outcomes and phenotypes					
Lund-Mackay CT score	14.69 ± 2.44	15.58 ± 2.54	14.75 ± 2.08	13.73 ± 2.34	*p* < 0.001
SNOT-22 score	50.57 ± 8.02	51.55 ± 8.96	50.52 ± 7.83	49.62 ± 7.11	*p* = 0.195
Peripheral eosinophils, 10^9/L	0.40 ± 0.11	0.40 ± 0.10	0.40 ± 0.12	0.39 ± 0.12	*p* = 0.662
High Lund-Mackay burden	113 (33.2)	52 (45.6)	38 (33.6)	23 (20.4)	*p* < 0.001
High SNOT-22 burden	113 (33.2)	43 (37.7)	38 (33.6)	32 (28.3)	*p* = 0.321
CRSwNP phenotype	161 (47.4)	58 (50.9)	54 (47.8)	49 (43.4)	*p* = 0.523
Elevated eosinophils	112 (32.9)	37 (32.5)	44 (38.9)	31 (27.4)	*p* = 0.182
Comorbidities and treatment characteristics					
Allergic rhinitis	136 (40.0)	53 (46.5)	41 (36.3)	42 (37.2)	*p* = 0.220
Asthma	54 (15.9)	15 (13.2)	20 (17.7)	19 (16.8)	*p* = 0.611
Diabetes	29 (8.5)	11 (9.6)	8 (7.1)	10 (8.8)	*p* = 0.778
Hypertension	54 (15.9)	19 (16.7)	16 (14.2)	19 (16.8)	*p* = 0.828
Antidiabetic drug use	15 (4.4)	8 (7.0)	3 (2.7)	4 (3.5)	*p* = 0.239
Intranasal corticosteroid use	177 (52.1)	62 (54.4)	55 (48.7)	60 (53.1)	*p* = 0.665

Values are mean ± SD or *n* (%). *p* values compare CDAI tertiles. CDAI, composite dietary antioxidant index; CRS, chronic rhinosinusitis; HOMA-IR, homeostatic model assessment of insulin resistance; SNOT-22, 22-item Sino-Nasal Outcome Test; TyG, triglyceride-glucose index.

**Figure 3:**
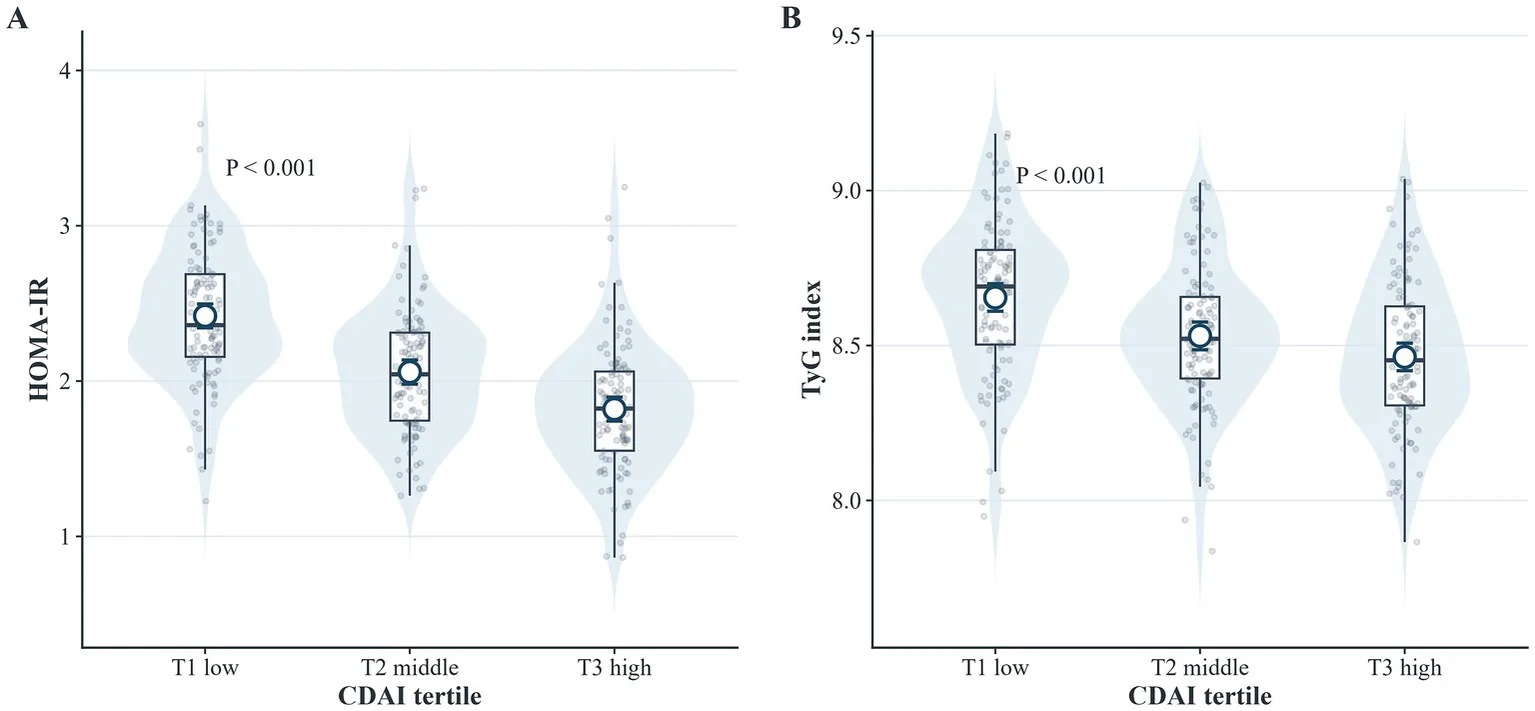
HOMA-IR and TyG index across CDAI tertiles. Panel **(A)** shows HOMA-IR, and Panel **(B)** shows TyG index. Violin plots, individual points, and boxplots show unadjusted distributions. White circles and error bars show adjusted means and 95% confidence intervals from models adjusted for age, sex, BMI, smoking, alcohol use, total energy intake, allergic rhinitis, asthma, CRSwNP phenotype, diabetes, hypertension, and intranasal corticosteroid use. *p* values indicate adjusted trends across CDAI tertiles.

Detailed CRS phenotype, inflammatory characteristics, and treatment exposure are summarized in Supplementary Table S4. CRSwNP phenotype, peripheral eosinophil counts, elevated peripheral eosinophils, disease duration, antihistamine use, leukotriene modifier use, biologic therapy, and long-term intranasal corticosteroid use were generally comparable across CDAI tertiles. Consistent with the baseline pattern, adjusted distributions of insulin resistance-related indicators showed stepwise decreases in HOMA-IR and TyG index across CDAI tertiles (Figure 3). In multivariable models, each 1-SD increment in standardized CDAI was strongly associated with lower HOMA-IR and lower TyG index (Supplementary Table S5 and Supplementary Figure S1). Supplementary metabolic-variable diagnostics showed high correlations between insulin resistance-related indicators and their component variables, no severe multicollinearity in the expanded model including waist circumference and HDL-C, and a stable CDAI-Lund-Mackay association after additional adjustment for waist circumference and HDL-C (Supplementary Table S6).

### Association of CDAI with continuous CRS burden outcomes

The association of CDAI with continuous CRS burden outcomes is summarized in Table 2. In fully adjusted models, each 1-SD increment in standardized CDAI was associated with a lower Lund-Mackay CT score (*β*, −0.799; 95% CI, −1.069 to −0.528; *p* < 0.001). Compared with T1, T2 was associated with a lower Lund-Mackay score (*β*, −0.967; 95% CI, −1.585 to −0.350), and T3 showed a stronger association (*β*, −1.876; 95% CI, −2.512 to −1.240). The trend across CDAI tertiles was significant.

**Table 2:** Association of CDAI with continuous CRS burden outcomes.

Outcome	Model 1	Model 2	Model 3
Lund-Mackay CT score			
CDAI per 1-SD increment	−0.782 (−1.055, −0.508); *P* < 0.001	−0.789 (−1.061, −0.518); *p* < 0.001	−0.799 (−1.069, −0.528); *p* < 0.001
CDAI T1 low	Reference	Reference	Reference
CDAI T2 middle	−0.858 (−1.469, −0.248); *p* = 0.006	−0.883 (−1.495, −0.271); *p* = 0.005	−0.967 (−1.585, −0.350); *p* = 0.002
CDAI T3 high	−1.835 (−2.475, −1.194); *p* < 0.001	−1.839 (−2.480, −1.199); *p* < 0.001	−1.876 (−2.512, −1.240); *p* < 0.001
P for trend across tertiles	*p* < 0.001	*p* < 0.001	*p* < 0.001
SNOT-22 score			
CDAI per 1-SD increment	−1.008 (−1.877, −0.139); *p* = 0.024	−0.985 (−1.851, −0.119); *p* = 0.027	−0.979 (−1.866, −0.093); *p* = 0.031
CDAI T1 low	Reference	Reference	Reference
CDAI T2 middle	−0.983 (−3.180, 1.213); *p* = 0.381	−1.008 (−3.214, 1.197); *p* = 0.371	−1.150 (−3.380, 1.081); *p* = 0.313
CDAI T3 high	−1.926 (−4.038, 0.186); *p* = 0.075	−1.862 (−3.985, 0.261); *p* = 0.087	−1.912 (−4.085, 0.262); *p* = 0.086
P for trend across tertiles	*p* = 0.074	*p* = 0.086	*p* = 0.085

Model 1 adjusted for age and sex. Model 2 further adjusted for BMI, current smoking, current alcohol use, and total energy intake. Model 3 further adjusted for allergic rhinitis, asthma, CRSwNP phenotype, diabetes, hypertension, and intranasal corticosteroid use. All principal regression models were fitted in the final analytic sample *n* = 340.

For SNOT-22 score, each 1-SD increment in standardized CDAI was associated with a modestly lower score in the fully adjusted model (*β*, −0.979; 95% CI, −1.866 to −0.093; *p* = 0.031). However, tertile-based estimates for SNOT-22 were weaker than those for Lund-Mackay and did not show a statistically significant trend. Adjusted prediction curves demonstrated a near-linear inverse association between CDAI and Lund-Mackay and a weaker inverse association between CDAI and SNOT-22 (Figure 2). Restricted cubic spline analyses showed no evidence of nonlinearity for Lund-Mackay CT score or SNOT-22 score (P for nonlinearity = 0.794 and 0.543, respectively). Model diagnostics for the fully adjusted linear regression models are summarized in Supplementary Table S7. The Lund-Mackay model showed mild residual non-normality by the Shapiro–Wilk test, whereas no significant heteroscedasticity or highly influential observations were identified in either model.

### Associations of insulin resistance indicators with Lund-Mackay outcomes

Higher insulin resistance-related indicator levels were associated with greater radiologic CRS burden. In fully adjusted models, HOMA-IR was positively associated with Lund-Mackay CT score and with high Lund-Mackay burden; similar positive associations were observed for TyG (Supplementary Table S8 and Supplementary Figure S2).

### Mediation through HOMA-IR and TyG index

**Figure 4:**
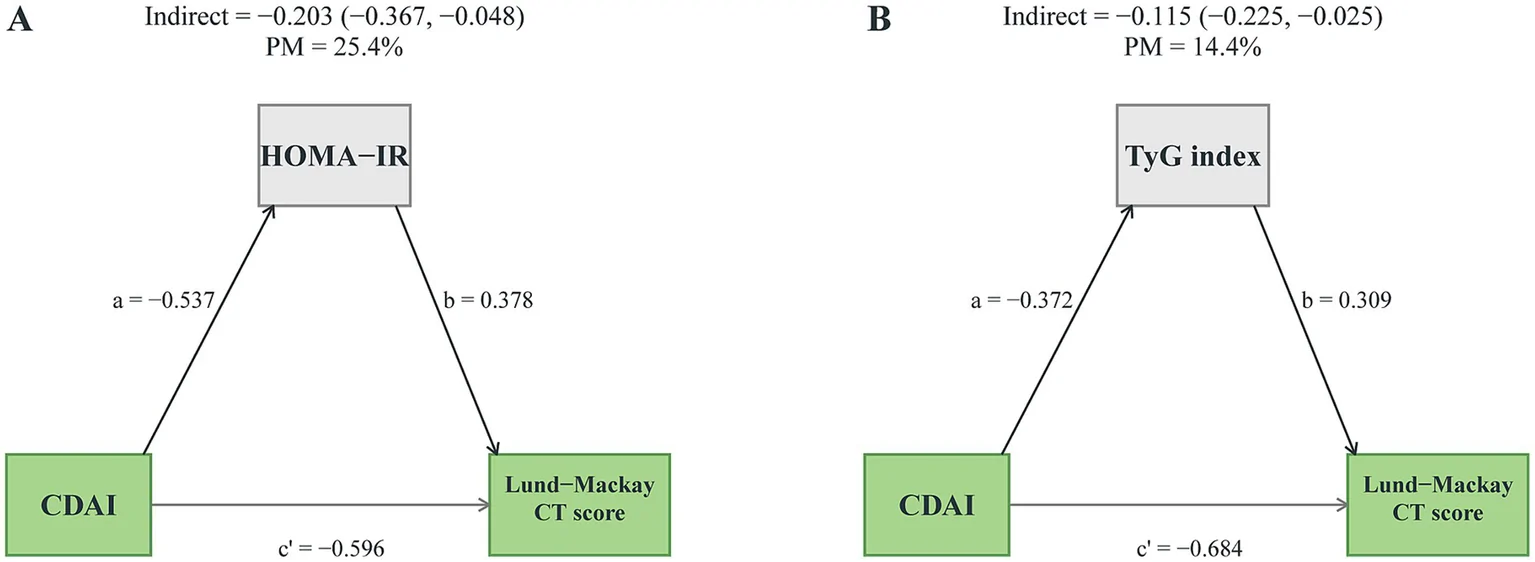
Exploratory mediation analysis of the CDAI-Lund-Mackay association through insulin resistance. Panel **(A)** shows the mediation pathway through HOMA-IR. Panel **(B)** shows the mediation pathway through TyG index. PM indicates proportion mediated. Path coefficients are unstandardized estimates, except that CDAI is expressed per 1-SD increment.

Exploratory mediation analyses are presented in Table 3 and Figure 4. The total association of CDAI with Lund-Mackay score was −0.799. HOMA-IR accounted for part of this association, with an indirect effect of −0.203 (95% CI, −0.367 to −0.048; *p* = 0.013), corresponding to a mediated proportion of 25.4%. TyG showed a smaller indirect component, with an indirect effect of −0.115 (95% CI, −0.225 to −0.025; *p* = 0.013), corresponding to a mediated proportion of 14.4%.

**Table 3:** Exploratory mediation analysis using HOMA-IR and TyG.

Mediator	Total effect	Direct effect	Indirect effect	Proportion mediated, %	P indirect
HOMA-IR	−0.799	−0.596	−0.203 (−0.367, −0.048)	25.4 (6.0, 51.0)	p = 0.013
TyG index	−0.799	−0.684	−0.115 (−0.225, −0.025)	14.4 (3.0, 30.5)	*p* = 0.013

Exploratory mediation analyses used CDAI z-score as the exposure and Lund-Mackay CT score as the outcome. HOMA-IR and TyG were modeled separately. The mediator and outcome models were adjusted for the same covariates as the fully adjusted model. Indirect effects and proportions mediated were estimated using 1,000 nonparametric bootstrap resamples, and percentile 95% confidence intervals are reported. Effects are unstandardized estimates, except that CDAI is expressed per 1-SD increment. Each exploratory mediation model included 340 participants. Given the cross-sectional design, these estimates should be interpreted as exploratory. TyG, triglyceride-glucose index.

### Sensitivity and subgroup analyses

Binary sensitivity analyses used upper-tertile definitions for high-burden outcomes. High Lund-Mackay burden was defined as Lund-Mackay CT score ≥16.006, and high SNOT-22 burden was defined as SNOT-22 score ≥53.508. Each 1-SD increment in standardized CDAI was associated with lower odds of high Lund-Mackay burden in the fully adjusted model (OR, 0.59; 95% CI, 0.45 to 0.77; *p* < 0.001). The association with high SNOT-22 burden was directionally consistent but weaker and not statistically significant (Supplementary Table S9). Restricted analyses excluding diabetes, antidiabetic drug use, supplement use, current smoking, or intranasal corticosteroid use showed broadly consistent CDAI-Lund-Mackay associations and preserved the direction of HOMA-IR and TyG indirect-effect estimates (Supplementary Table S10).

**Figure 5:**
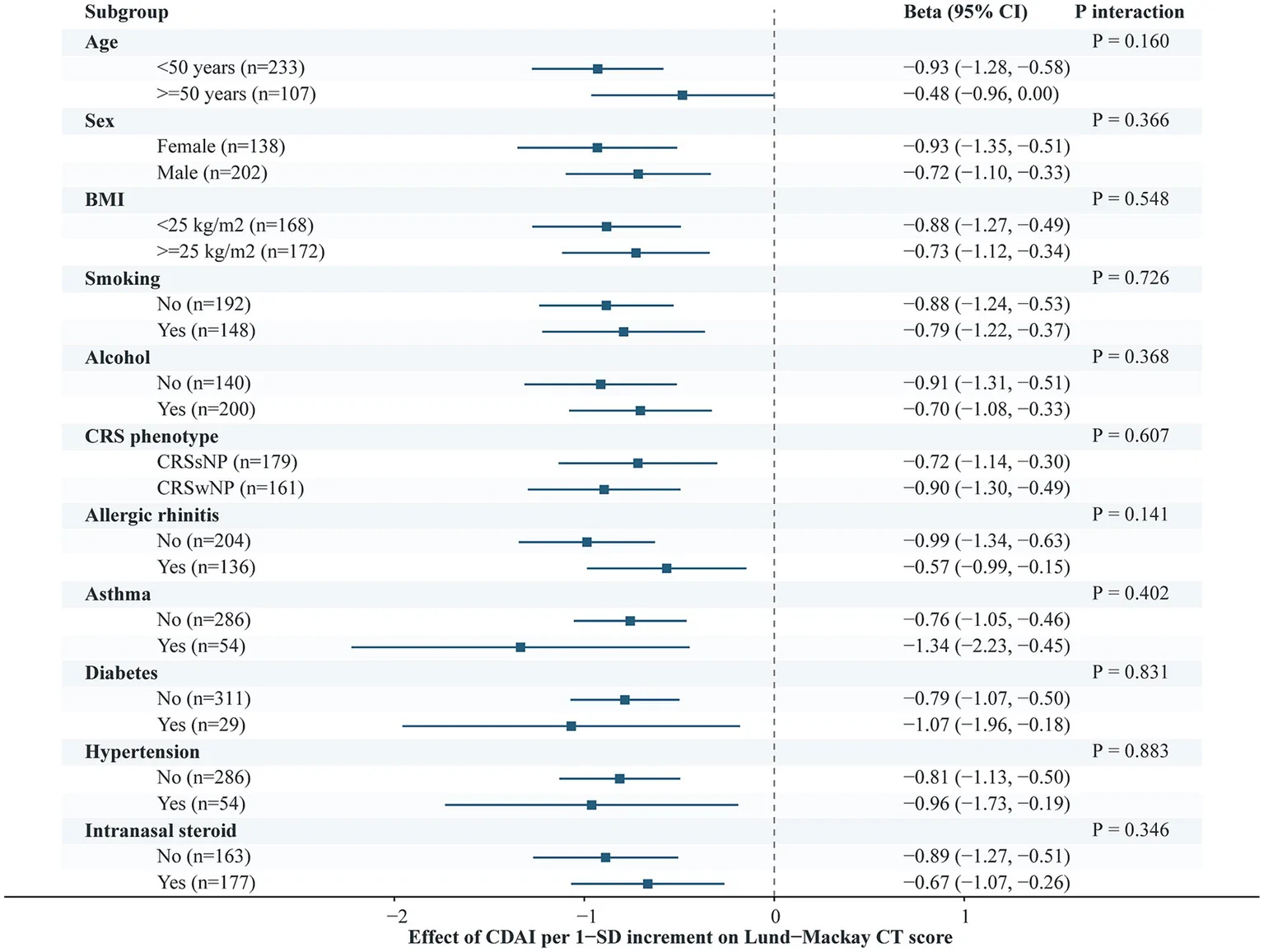
Subgroup analysis of the association between CDAI and Lund-Mackay CT score. Forest plot showing *β* coefficients and 95% confidence intervals for the association of standardized CDAI per 1-SD increment with Lund-Mackay CT score across prespecified subgroups. *p* values represent interaction tests.

Subgroup analyses showed inverse associations between CDAI and Lund-Mackay score across strata of age, sex, BMI, smoking, alcohol use, CRS phenotype, allergic rhinitis, asthma, diabetes, hypertension, and intranasal corticosteroid use. No statistically significant interaction was observed across the prespecified subgroups (Figure 5 and Supplementary Table S11).

## Discussion

In this cross-sectional analysis of prospectively collected baseline data from adults with CRS, higher CDAI score was associated with lower radiologic disease burden as assessed by Lund-Mackay CT score. The association remained stable after adjustment for demographic, lifestyle, CRS-related, and metabolic covariates. CDAI also showed an inverse association with SNOT-22 score when modeled continuously, although the tertile-based results for SNOT-22 were weaker than those for Lund-Mackay, supporting SNOT-22 as a secondary supportive outcome rather than the primary mechanistic endpoint. The exploratory mediation analyses further suggested that insulin resistance may partly account for the association between CDAI and radiologic CRS burden. HOMA-IR accounted for a larger proportion of the association than TyG, consistent with its direct incorporation of fasting glucose and insulin. TyG nevertheless provided supportive evidence as a non-insulin surrogate measure that may be easier to obtain in routine clinical settings. Sensitivity and subgroup analyses showed generally consistent directions, strengthening the internal coherence of the findings.

The present findings extend current CRS research in several ways. Contemporary CRS guidelines and consensus statements emphasize the heterogeneity of CRS and the need to move beyond an infection-centered model toward inflammatory, immune, and treatable-trait frameworks (1, 2). Prior work has established the substantial epidemiologic and quality-of-life burden of CRS, but modifiable systemic nutritional and metabolic factors have received comparatively less attention (26, 27). By linking CDAI with CT-defined disease burden, the current analysis adds a dietary and metabolic dimension to CRS phenotyping. Our results are also consistent with literature showing that oxidative stress and epithelial barrier dysfunction are relevant to CRS pathophysiology (28). Recent studies have highlighted epithelial oxidative injury, reactive oxygen species-related signaling, and inflammasome activation in CRS and CRSwNP inflammation (29, 30). Although the present study did not measure local sinonasal oxidative biomarkers, the observed inverse association between CDAI and radiologic disease burden is directionally compatible with these mechanistic observations.

From a nutritional epidemiology perspective, the findings align with recent studies linking higher CDAI to more favorable cardiometabolic profiles, including lower diabetes and metabolic syndrome burden (12). The inverse association between CDAI and both HOMA-IR and TyG also agrees with emerging evidence that composite antioxidant dietary patterns are related to insulin resistance-related indices (14). These studies collectively suggest that CDAI may capture a broader diet-metabolism signal than isolated antioxidant nutrients.

Several mechanisms may explain the observed association. Antioxidant micronutrients included in CDAI, such as vitamins A, C, and E, carotenoids, zinc, and selenium, participate in redox regulation, epithelial integrity, innate immune function, and inflammatory balance. In CRS, oxidative stress may impair tight junctions, enhance epithelial permeability, and facilitate persistent mucosal inflammation (31). A higher composite antioxidant intake could therefore reflect a dietary milieu less permissive to oxidative epithelial injury and inflammatory amplification. Among the individual CDAI components, selenium may be particularly relevant to the antioxidant-inflammatory context. Selenium is incorporated into selenoproteins involved in antioxidant defense and redox homeostasis, and may also participate in inflammatory signaling and immune regulation (32). Recent evidence from another chronic inflammatory condition reported an association between dietary selenium intake and endometriosis risk, further supporting the relevance of selenium-related oxidative-stress and immune mechanisms in inflammatory disease contexts (33). Taken together, these findings support the biological plausibility of selenium-related redox and immune regulation in chronic inflammatory settings.

The clinical interpretation of these findings should be considered in the context of a baseline observational study. The fully adjusted association of approximately 0.8 Lund-Mackay points per 1-SD increment in standardized CDAI represents a modest difference at the individual level, whereas the difference between the highest and lowest CDAI tertiles approached 1.9 points. Because the Lund-Mackay system scores opacification across multiple sinus regions, this magnitude may represent a measurable population-level difference in objective radiologic inflammatory burden. Previous work has shown that Lund-Mackay score captures a disease dimension not represented by symptom scores alone and is related to clinically relevant indicators, including polyp grade, the extent of surgery offered, postoperative symptom reduction, complication rates, and revision rates (34). Therefore, the observed association may be clinically informative for CRS phenotyping, objective burden stratification, and identification of systemic factors associated with more severe radiologic disease.

The weaker association with SNOT-22 is also clinically plausible. Dietary antioxidant capacity and insulin resistance-related metabolism may be more closely linked to oxidative stress, low-grade inflammation, epithelial dysfunction, mucosal edema, and inflammatory opacification, which are more directly reflected by CT-based Lund-Mackay scoring. By contrast, SNOT-22 is a broad patient-reported outcome influenced by sinonasal symptoms as well as sleep disturbance, emotional status, pain perception, olfactory dysfunction, and comorbid airway disease (35). Recent evidence in CRSwNP further supports a relative disconnect between SNOT-22 and objective endoscopic/radiologic measures, including nasal polyp score and Lund-Mackay score (36). Thus, the small SNOT-22 association should be interpreted as supportive rather than clinically strong, while the overall pattern suggests that CDAI may be more closely related to objective radiologic CRS burden than to immediate patient-perceived symptom burden. This supports the clinical relevance of a potentially modifiable dietary-metabolic phenotype and provides a rationale for future longitudinal and interventional studies.

Insulin resistance provides a biologically plausible link between antioxidant dietary capacity and CRS burden. Chronic insulin resistance is accompanied by low-grade inflammation, oxidative stress, adipokine dysregulation, and altered innate and adaptive immune responses (37). These processes may influence sinonasal mucosal inflammation indirectly through systemic inflammatory priming, endothelial and epithelial dysfunction, or impaired resolution of inflammation. Insulin resistance is unlikely to be the only metabolic-inflammatory process underlying the observed association. Central adiposity and systemic inflammatory markers may represent additional or overlapping mechanisms linking dietary antioxidant capacity with CRS burden. Evidence from another chronic inflammatory disease showed that central adiposity indices and inflammatory markers statistically mediated the association between an overall cardiovascular health metric and periodontitis, supporting the relevance of adiposity-inflammatory pathways in chronic inflammatory disease burden (38). CRS-specific studies have also reported obesity-related cytokine differences and distinct peripheral blood metabolic profiles in patients with CRS, suggesting that CRS may involve systemic metabolic-inflammatory alterations (39, 40). In the present study, waist circumference was available and was considered in an expanded metabolic-adjustment sensitivity model, in which the CDAI-Lund-Mackay association remained stable. Further studies incorporating systemic inflammatory cytokines and local inflammatory biomarkers are warranted to clarify how adiposity-inflammatory mechanisms interact with insulin resistance-related metabolism in CRS.

The larger mediated proportion for HOMA-IR suggests that the fasting glucose-insulin axis may be more closely involved in the observed association between lower antioxidant dietary capacity and greater CT burden. HOMA-IR integrates fasting glucose and insulin and remains a classical epidemiologic marker of insulin resistance (41). In contrast, TyG relies on fasting triglycerides and glucose and may capture a related but partially distinct metabolic phenotype (42). The fact that both mediation models yielded significant indirect effects, with a larger estimate for HOMA-IR than for TyG, supports the interpretation that insulin resistance-related metabolism may be involved, while also suggesting a hierarchy between the primary mediator and the pragmatic surrogate. However, because HOMA-IR and TyG are correlated metabolic indices, these separate mediation models should not be interpreted as proving pathway specificity or independent biological mechanisms. Rather, the findings suggest that insulin resistance-related metabolism, as captured by complementary fasting metabolic indices, may be relevant to the observed CDAI-Lund-Mackay association.

TyG may still have practical value. Recent studies have supported TyG as a convenient metabolic risk marker that may outperform or complement HOMA-IR in some settings (43). Because fasting insulin is not routinely measured in many clinical environments, TyG could be useful for identifying patients in whom diet-related metabolic inflammation may be relevant to CRS burden. This point is especially important for future screening or risk-stratification studies, although clinical implementation should await prospective validation.

These findings should be interpreted as hypothesis-generating rather than practice-changing. The results do not prove that increasing CDAI will reduce CRS burden, nor do they imply that insulin resistance treatment will directly improve sinonasal inflammation. However, they suggest that dietary antioxidant capacity and insulin resistance-related status may help characterize a systemic vulnerability profile in CRS. This may be relevant when evaluating CRS patients with concomitant metabolic risk, obesity, diabetes, or lifestyle-related inflammatory burden.

Future studies should address several priorities. Longitudinal cohorts are needed to determine whether low CDAI precedes worsening radiologic burden or whether CRS severity influences dietary intake. Interventional studies could test whether dietary antioxidant improvement modifies insulin resistance and CRS outcomes. Mechanistic work incorporating sinonasal tissue, local oxidative stress biomarkers, epithelial barrier assays, and microbiome profiling would help clarify the biological processes underlying the observed mediation findings.

Several limitations warrant emphasis. First, the cross-sectional design precludes causal inference and cannot confirm temporal ordering among CDAI, insulin resistance, and CRS burden. Reverse or bidirectional associations also remain possible, because greater CRS burden may influence dietary intake, lifestyle behaviors, treatment exposure, and metabolic status. Second, dietary assessment remains vulnerable to recall error and reporting bias, although repeated recalls and energy adjustment were used to improve exposure assessment. Residual confounding may also persist from incompletely captured socioeconomic status, education, physical activity, overall dietary quality, lipid-lowering medication, and treatment history. In particular, lipid-lowering therapy may influence triglycerides and TyG, whereas antidiabetic treatment may influence fasting glucose, fasting insulin, and HOMA-IR. Future studies with more detailed lifestyle, dietary-quality, and medication data are warranted. Third, the single-center setting and eligibility criteria may limit generalizability to all CRS populations, particularly patients with milder disease, different dietary patterns, or distinct inflammatory endotypes. Fourth, although Lund-Mackay CT scores were independently assessed by two senior otolaryngologists with adjudication by a third otolaryngologist when needed, formal inter-rater reliability statistics were not calculated. Fifth, SNOT-22 may be affected by non-sinonasal factors such as sleep disturbance, mood, pain perception, and comorbid airway disease, which may partly explain the weaker symptom-based associations. In addition, although CRS phenotype, peripheral eosinophils, disease duration, and relevant treatment exposures were further described, standardized tissue eosinophil counts and standardized endoscopic scores were not systematically available for all participants. Residual heterogeneity related to local inflammatory endotype and endoscopic severity therefore cannot be fully excluded. Finally, the present analysis did not include local sinonasal oxidative stress markers, systemic inflammatory cytokines, microbiome data, or longitudinal response to therapy. Although waist circumference was considered in an expanded metabolic-adjustment sensitivity model, systemic inflammatory cytokines and other biomarkers needed to formally evaluate adiposity-inflammatory pathways were not systematically available. Therefore, the mediation findings should be interpreted as exploratory and hypothesis-generating, and they require confirmation in longitudinal and interventional studies.

## Conclusion

Higher CDAI score was associated with lower radiologic CRS burden in adults with CRS. Exploratory mediation analyses suggested that insulin resistance may partly account for this association, with HOMA-IR showing a larger contribution and TyG providing supportive evidence as a pragmatic non-insulin surrogate. These findings suggest that dietary antioxidant capacity and insulin resistance-related metabolic pathways may be relevant to CRS burden, but prospective and interventional studies are required to verify temporality and causality.

## Data Availability

The datasets presented in this article are not readily available due to institutional privacy restrictions. Requests to access the datasets should be directed to the corresponding author, upon reasonable request and with appropriate institutional approval.
