# Supplementary material for: Association between the composite dietary antioxidant index and chronic rhinosinusitis burden: the potential mediating role of insulin resistance
Source: Front Nutr. 2026 Aug 31;13:1908084. doi: 10.3389/fnut.2026.1908084 (PMC13572122; doi:10.3389/fnut.2026.1908084)
Supplement: Supplementary file 1 [file Table_1.DOCX]

**Supplementary Tables**

**Supplementary Table 1. Variable definitions and formulas**

| **Variable** | **Formula / definition** | **Analytical role** |
| --- | --- | --- |
| CDAI | Energy-adjusted composite dietary antioxidant index calculated from vitamin A, vitamin C, vitamin E, carotenoids, zinc, and selenium. | Primary exposure |
| Standardized CDAI | CDAI score rescaled to a mean of 0 and SD of 1. | Continuous exposure for per-SD regression and mediation analyses |
| HOMA-IR | FPG (mmol/L) x fasting insulin (μU/mL) / 22.5. | Primary mediator |
| TyG | ln[TG (mg/dL) x FPG (mg/dL) / 2]. | Pragmatic non-insulin mediator |
| Lund-Mackay CT score | CT-based CRS burden score ranging from 0 to 24. | Primary outcome |
| SNOT-22 score | Patient-reported symptom and quality-of-life score ranging from 0 to 110. | Secondary outcome |
| High Lund-Mackay burden | Binary indicator for Lund-Mackay CT score in the highest tertile of the score distribution in the final analytic sample. | Binary sensitivity outcome |
| High SNOT-22 burden | Binary indicator for SNOT-22 score in the highest tertile of the score distribution in the final analytic sample. | Binary sensitivity outcome |
| Recent systemic steroid | Any systemic corticosteroid use within 4 weeks before enrollment. | Exclusion criterion |
| Recent antibiotic | Any antibiotic use within 4 weeks before enrollment. | Exclusion criterion |

**Note.** CDAI, composite dietary antioxidant index; CRS, chronic rhinosinusitis; FPG, fasting plasma glucose; HOMA-IR, homeostatic model assessment of insulin resistance; SNOT-22, 22-item Sino-Nasal Outcome Test; TG, triglycerides; TyG, triglyceride-glucose index.

**Supplementary Table 2. Energy-adjusted antioxidant nutrient intake across CDAI tertiles**

| **Variable** | **Overall** | **T1 low** | **T2 middle** | **T3 high** | **P value** |
| --- | --- | --- | --- | --- | --- |
| Energy-adjusted Vitamin A, ug/day | 591.63 ± 143.39 | 454.50 ± 101.72 | 594.20 ± 84.66 | 727.42 ± 82.92 | P < 0.001 |
| Energy-adjusted Vitamin C, mg/day | 79.03 ± 26.32 | 53.36 ± 17.66 | 79.67 ± 13.82 | 104.31 ± 16.57 | P < 0.001 |
| Energy-adjusted Vitamin E, mg/day | 9.53 ± 2.59 | 7.05 ± 1.72 | 9.52 ± 1.14 | 12.06 ± 1.80 | P < 0.001 |
| Energy-adjusted Carotenoids, ug/day | 3473.45 ± 1013.01 | 2478.73 ± 617.16 | 3463.97 ± 496.70 | 4486.46 ± 657.61 | P < 0.001 |
| Energy-adjusted Zinc, mg/day | 9.22 ± 2.02 | 7.12 ± 1.20 | 9.36 ± 0.99 | 11.19 ± 1.20 | P < 0.001 |
| Energy-adjusted Selenium, ug/day | 54.76 ± 11.23 | 43.58 ± 6.25 | 54.46 ± 5.28 | 66.34 ± 7.22 | P < 0.001 |
| Total energy, kcal/day | 2108.18 ± 361.34 | 2079.80 ± 339.40 | 2165.19 ± 388.52 | 2079.80 ± 350.88 | P = 0.122 |

**Note.** Values are presented as mean ± SD. P values compare CDAI tertiles. CDAI tertiles were defined using the energy-adjusted composite dietary antioxidant index, with T1 representing the lowest tertile and T3 the highest tertile.

# **Supplementary Table 3. Distribution of CDAI and antioxidant nutrient components**

| **Variable** | **Unit** | **N** | **Mean** | **SD** | **Median** | **Minimum** | **Maximum** |
| --- | --- | --- | --- | --- | --- | --- | --- |
| Energy-adjusted Vitamin A | ug/day | 340 | 591.634 | 143.350 | 599.764 | 246.126 | 1094.384 |
| Energy-adjusted Vitamin C | mg/day | 340 | 79.034 | 26.256 | 79.316 | 23.089 | 150.263 |
| Energy-adjusted Vitamin E | mg/day | 340 | 9.534 | 2.585 | 9.589 | 3.997 | 16.380 |
| Energy-adjusted Carotenoids | ug/day | 340 | 3473.451 | 1012.571 | 3478.713 | 1167.704 | 6223.872 |
| Energy-adjusted Zinc | mg/day | 340 | 9.220 | 2.015 | 9.353 | 4.911 | 15.012 |
| Energy-adjusted Selenium | ug/day | 340 | 54.760 | 11.233 | 54.057 | 24.929 | 88.496 |
| CDAI score | unitless | 340 | 0.000 | 5.353 | -0.152 | -13.236 | 17.331 |
| Standardized CDAI | unitless | 340 | 0.000 | 1.000 | -0.028 | -2.473 | 3.238 |

**Note**. Values were calculated in the final analytic sample. Each antioxidant nutrient was energy-adjusted using the residual method before standardization. CDAI was calculated as the sum of the six study-specific standardized z-scores for vitamin A, vitamin C, vitamin E, carotenoids, zinc, and selenium. Because CDAI was constructed from study-specific z-scores, its mean is centered around zero. The final CDAI score was additionally standardized for per-SD regression analyses. No external reference distribution was used for CDAI standardization. CDAI, composite dietary antioxidant index; SD, standard deviation.

**Supplementary Table 4. Detailed CRS phenotype, inflammatory characteristics, and treatment exposure according to CDAI tertiles**

| **Variable** | **Overall** | **T1 low** | **T2 middle** | **T3 high** | **P value** |
| --- | --- | --- | --- | --- | --- |
| CRSwNP phenotype | 161 (47.4) | 58 (50.9) | 54 (47.8) | 49 (43.4) | P = 0.523 |
| Peripheral eosinophils, 10^9/L | 0.40 ± 0.11 | 0.40 ± 0.10 | 0.40 ± 0.12 | 0.39 ± 0.12 | P = 0.662 |
| Elevated peripheral eosinophils | 112 (32.9) | 37 (32.5) | 44 (38.9) | 31 (27.4) | P = 0.182 |
| Disease duration, months | 38.9 ± 20.6 | 40.2 ± 21.5 | 38.4 ± 20.3 | 38.0 ± 19.9 | P = 0.704 |
| Antihistamine use | 122 (35.9) | 44 (38.6) | 40 (35.4) | 38 (33.6) | P = 0.724 |
| Leukotriene modifier use | 66 (19.4) | 24 (21.1) | 22 (19.5) | 20 (17.7) | P = 0.805 |
| Biologic therapy | 3 (0.9) | 1 (0.9) | 1 (0.9) | 1 (0.9) | P = 1.000 |
| Long-term intranasal corticosteroid use | 177 (52.1) | 62 (54.4) | 55 (48.7) | 60 (53.1) | P = 0.665 |

**Note.** Values are mean ± SD or n (%). P values compare CDAI tertiles. All included patients had not undergone prior sinus surgery; therefore, prior sinus surgery was not tabulated as a separate baseline variable. Peripheral eosinophil counts were reported as a systemic inflammatory marker. Elevated peripheral eosinophils were defined according to the institutional laboratory reference range. Standardized tissue eosinophil counts and standardized endoscopic scores were not systematically available for all participants. CDAI, composite dietary antioxidant index; CRS, chronic rhinosinusitis; CRSwNP, chronic rhinosinusitis with nasal polyps.

**Supplementary Table 5. CDAI and HOMA-IR/TyG**

| **Mediator** | **Model 1** | **Model 2** | **Model 3** |
| --- | --- | --- | --- |
| HOMA-IR | -0.535 (-0.618, -0.451); P < 0.001 | -0.536 (-0.620, -0.452); P < 0.001 | -0.537 (-0.623, -0.452); P < 0.001 |
| TyG index | -0.371 (-0.465, -0.278); P < 0.001 | -0.373 (-0.467, -0.279); P < 0.001 | -0.372 (-0.468, -0.276); P < 0.001 |

**Note.** Values are beta coefficients (95% CIs) for the association of CDAI per 1-SD increment with insulin resistance-related indicators. Model 1 adjusted for age and sex. Model 2 further adjusted for BMI, current smoking, current alcohol use, and total energy intake. Model 3 further adjusted for allergic rhinitis, asthma, CRSwNP phenotype, diabetes, hypertension, and intranasal corticosteroid use.

**Supplementary Table 6. Metabolic-variable diagnostics supporting covariate selection**

| Diagnostic item | Result | Interpretation |
| --- | --- | --- |
| Spearman correlation between BMI and waist circumference | r = 0.42 | Moderate correlation between adiposity-related measures |
| Spearman correlation between HOMA-IR and fasting insulin | r = 0.97 | High overlap because fasting insulin is a component of HOMA-IR |
| Spearman correlation between TyG index and triglycerides | r = 0.98 | High overlap because triglycerides are a component of TyG |
| Maximum VIF in the expanded model including waist circumference and HDL-C | 1.83 | No evidence of severe multicollinearity in the expanded model |
| Standardized CDAI in the primary fully adjusted model | β = -0.799 (95% CI: -1.043 to -0.554); P < 0.001 | Primary fully adjusted association with Lund-Mackay CT score |
| Standardized CDAI after additional adjustment for waist circumference and HDL-C | β = -0.858 (95% CI: -1.099 to -0.617); P < 0.001 | Association remained stable after expanded metabolic adjustment |

**Note.** Spearman correlations were calculated in the final analytic sample (n = 340). The expanded model was based on the primary fully adjusted model and additionally included waist circumference and HDL-C. VIF values were calculated in the expanded model. Regression estimates are beta coefficients and 95% confidence intervals for the association of standardized CDAI per 1-SD increment with Lund-Mackay CT score. BMI, body mass index; CDAI, composite dietary antioxidant index; CI, confidence interval; HDL-C, high-density lipoprotein cholesterol; HOMA-IR, homeostatic model assessment of insulin resistance; TyG, triglyceride-glucose index; VIF, variance inflation factor.

**Supplementary Table 7. Diagnostic assessment of the fully adjusted linear regression models**

| **Diagnostic domain** | **Lund-Mackay CT score model** | **SNOT-22 score model** |
| --- | --- | --- |
| Shapiro-Wilk test | W = 0.991; P = 0.030 | W = 0.993; P = 0.142 |
| Breusch-Pagan test | P = 0.668 | P = 0.095 |
| Maximum Cook's distance | 0.036 | 0.040 |
| Cook's distance > 1 | 0 | 0 |

**Note.** Model diagnostics were performed for the fully adjusted linear regression models. The Lund-Mackay model showed mild residual non-normality by the Shapiro-Wilk test, whereas no significant heteroscedasticity or highly influential observations were identified in either model. BMI, body mass index; CDAI, composite dietary antioxidant index; CRSwNP, chronic rhinosinusitis with nasal polyps; SNOT-22, 22-item Sino-Nasal Outcome Test.

**Supplementary Table 8. HOMA-IR/TyG and Lund-Mackay outcomes**

| **Mediator** | **Outcome** | **Model 1** | **Model 2** | **Model 3** |
| --- | --- | --- | --- | --- |
| HOMA-IR | Lund-Mackay CT score | 0.709 (0.440, 0.978); P < 0.001 | 0.709 (0.438, 0.980); P < 0.001 | 0.701 (0.438, 0.965); P < 0.001 |
| HOMA-IR | High Lund-Mackay burden | 2.14 (1.62, 2.82); P < 0.001 | 2.17 (1.63, 2.89); P < 0.001 | 2.20 (1.65, 2.92); P < 0.001 |
| TyG index | Lund-Mackay CT score | 0.555 (0.301, 0.809); P < 0.001 | 0.548 (0.289, 0.807); P < 0.001 | 0.564 (0.311, 0.816); P < 0.001 |
| TyG index | High Lund-Mackay burden | 1.55 (1.22, 1.98); P < 0.001 | 1.55 (1.21, 1.99); P < 0.001 | 1.60 (1.24, 2.06); P < 0.001 |

**Note.** For Lund-Mackay CT score, values are beta coefficients (95% CIs). For high Lund-Mackay burden, values are odds ratios (95% CIs). Model 1 adjusted for age and sex. Model 2 further adjusted for BMI, current smoking, current alcohol use, and total energy intake. Model 3 further adjusted for allergic rhinitis, asthma, CRSwNP phenotype, diabetes, hypertension, and intranasal corticosteroid use. High Lund-Mackay burden was defined as the highest tertile of Lund-Mackay CT score.

**Supplementary Table 9. Binary outcome sensitivity analyses**

| **Outcome** | **Exposure** | **Model 1** | **Model 2** | **Model 3** |
| --- | --- | --- | --- | --- |
| High Lund-Mackay burden | CDAI per 1-SD increment | 0.60 (0.47, 0.78); P < 0.001 | 0.60 (0.47, 0.78); P < 0.001 | 0.59 (0.45, 0.77); P < 0.001 |
| High Lund-Mackay burden | CDAI T1 low | Reference | Reference | Reference |
| High Lund-Mackay burden | CDAI T2 middle | 0.60 (0.35, 1.03); P = 0.065 | 0.60 (0.34, 1.05); P = 0.075 | 0.57 (0.32, 1.01); P = 0.053 |
| High Lund-Mackay burden | CDAI T3 high | 0.31 (0.17, 0.56); P < 0.001 | 0.31 (0.17, 0.56); P < 0.001 | 0.29 (0.15, 0.55); P < 0.001 |
| High Lund-Mackay burden | P for trend across tertiles | P < 0.001 | P < 0.001 | P < 0.001 |
| High SNOT-22 burden | CDAI per 1-SD increment | 0.81 (0.64, 1.03); P = 0.081 | 0.82 (0.64, 1.04); P = 0.107 | 0.82 (0.64, 1.06); P = 0.130 |
| High SNOT-22 burden | CDAI T1 low | Reference | Reference | Reference |
| High SNOT-22 burden | CDAI T2 middle | 0.84 (0.48, 1.47); P = 0.546 | 0.84 (0.47, 1.48); P = 0.544 | 0.83 (0.46, 1.50); P = 0.543 |
| High SNOT-22 burden | CDAI T3 high | 0.65 (0.37, 1.15); P = 0.142 | 0.68 (0.38, 1.22); P = 0.198 | 0.68 (0.37, 1.26); P = 0.221 |
| High SNOT-22 burden | P for trend across tertiles | P = 0.141 | P = 0.197 | P = 0.220 |

**Note.** Values are odds ratios (95% CIs) for binary outcomes unless otherwise indicated. High Lund-Mackay burden was defined as Lund-Mackay CT score in the highest tertile of the score distribution in the final analytic sample. High SNOT-22 burden was defined as SNOT-22 score in the highest tertile of the score distribution in the final analytic sample. Model 1 adjusted for age and sex. Model 2 further adjusted for BMI, current smoking, current alcohol use, and total energy intake. Model 3 further adjusted for allergic rhinitis, asthma, CRSwNP phenotype, diabetes, hypertension, and intranasal corticosteroid use. All binary sensitivity models were fitted in the final analytic sample (n = 340).

**Supplementary Table 10. Restricted sensitivity analyses**

| **Analysis** | **n** | **CDAI-Lund-Mackay beta** | **HOMA-IR indirect effect (proportion)** | **TyG indirect effect (proportion)** |
| --- | --- | --- | --- | --- |
| Main analysis | 340 | -0.799 (-1.069, -0.528); P < 0.001 | -0.203 (25.4%) | -0.115 (14.4%) |
| Excluding diabetes | 311 | -0.786 (-1.073, -0.499); P < 0.001 | -0.179 (22.8%) | -0.115 (14.6%) |
| Excluding antidiabetic drug use | 325 | -0.788 (-1.068, -0.508); P < 0.001 | -0.188 (23.8%) | -0.118 (15.0%) |
| Excluding supplement users | 295 | -0.853 (-1.133, -0.573); P < 0.001 | -0.167 (19.6%) | -0.118 (13.9%) |
| Excluding current smokers | 192 | -0.884 (-1.238, -0.531); P < 0.001 | -0.259 (29.3%) | -0.113 (12.8%) |
| Excluding intranasal steroid users | 163 | -0.888 (-1.269, -0.506); P < 0.001 | -0.147 (16.6%) | -0.106 (11.9%) |

**Note.** CDAI-Lund-Mackay beta represents the fully adjusted beta coefficient for the association of CDAI per 1-SD increment with Lund-Mackay CT score. Indirect effects were estimated from mediation analyses; proportions mediated are shown in parentheses. Restricted analyses used the same modeling framework within each analytic subset.

**Supplementary Table 11. Subgroup analyses**

| **Group** | **Level** | **Beta (95% CI)** | **P interaction** |
| --- | --- | --- | --- |
| Age | <50 years (n=233) | -0.93 (-1.28, -0.58) | P = 0.160 |
| Age | ≥50 years (n=107) | -0.48 (-0.96, 0.00) | P = 0.160 |
| Sex | Female (n=138) | -0.93 (-1.35, -0.51) | P = 0.366 |
| Sex | Male (n=202) | -0.72 (-1.10, -0.33) | P = 0.366 |
| BMI | <25 kg/m² (n=168) | -0.88 (-1.27, -0.49) | P = 0.548 |
| BMI | ≥25 kg/m² (n=172) | -0.73 (-1.12, -0.34) | P = 0.548 |
| Smoking | No (n=192) | -0.88 (-1.24, -0.53) | P = 0.726 |
| Smoking | Yes (n=148) | -0.79 (-1.22, -0.37) | P = 0.726 |
| Alcohol | No (n=140) | -0.91 (-1.31, -0.51) | P = 0.368 |
| Alcohol | Yes (n=200) | -0.70 (-1.08, -0.33) | P = 0.368 |
| CRS phenotype | CRSsNP (n=179) | -0.72 (-1.14, -0.30) | P = 0.607 |
| CRS phenotype | CRSwNP (n=161) | -0.90 (-1.30, -0.49) | P = 0.607 |
| Allergic rhinitis | No (n=204) | -0.99 (-1.34, -0.63) | P = 0.141 |
| Allergic rhinitis | Yes (n=136) | -0.57 (-0.99, -0.15) | P = 0.141 |
| Asthma | No (n=286) | -0.76 (-1.05, -0.46) | P = 0.402 |
| Asthma | Yes (n=54) | -1.34 (-2.23, -0.45) | P = 0.402 |
| Diabetes | No (n=311) | -0.79 (-1.07, -0.50) | P = 0.831 |
| Diabetes | Yes (n=29) | -1.07 (-1.96, -0.18) | P = 0.831 |
| Hypertension | No (n=286) | -0.81 (-1.13, -0.50) | P = 0.883 |
| Hypertension | Yes (n=54) | -0.96 (-1.73, -0.19) | P = 0.883 |
| Intranasal steroid | No (n=163) | -0.89 (-1.27, -0.51) | P = 0.346 |
| Intranasal steroid | Yes (n=177) | -0.67 (-1.07, -0.26) | P = 0.346 |

**Note.** Values are beta coefficients (95% CIs) for the association of CDAI per 1-SD increment with Lund-Mackay CT score within each subgroup. P interaction values were obtained from models including a multiplicative interaction term between CDAI and the subgroup variable. BMI, body mass index; CRSsNP, chronic rhinosinusitis without nasal polyps; CRSwNP, chronic rhinosinusitis with nasal polyps.

**Supplementary Figures**

**Supplementary Figure 1. Adjusted associations of CDAI with insulin resistance-related indicators.**
Panel A shows the adjusted association between standardized CDAI and HOMA-IR. Panel B shows the adjusted association between standardized CDAI and the triglyceride-glucose (TyG) index. Solid lines represent adjusted predicted values, and shaded areas represent 95% confidence intervals.

**Supplementary Figure 2. Adjusted associations of insulin resistance-related indicators with Lund-Mackay CT score.**
Panel A shows the adjusted association between HOMA-IR and Lund-Mackay CT score. Panel B shows the adjusted association between the triglyceride-glucose (TyG) index and Lund-Mackay CT score. Solid lines represent adjusted predicted values, and shaded areas represent 95% confidence intervals.
